# Hemodynamic study of TCPC using in vivo and in vitro 4D flow MRI and numerical simulation

**DOI:** 10.1186/1532-429X-16-S1-W39

**Published:** 2014-01-16

**Authors:** Alejandro Roldán-Alzate, Sylvana García-Rodríguez, Leonardo Rivera, Oliver Wieben, Petros V Anagnostopoulos, Christopher J Francois

**Affiliations:** 1 Radiology, University of Wisconsin, Madison, Wisconsin, USA; 2 Physics, University of Wisconsin, Madison, Wisconsin, USA; 3 Medical Physics, University of Wisconsin, Madison, Wisconsin, USA; 4 Pediatric Cardiothoracic Surgery, University of Wisconsin, Madison, Wisconsin, USA

## Background

Altered total cavo-pulmonary connection (TCPC) hemodynamics results in numerous complications, including decreased exercise capacity, arrhythmia and ventricular failure. Patient-specific anatomy hinders a general solution. MRI is increasingly being used to study TCPC flow. However, it cannot predict behavior under extreme conditions or determine surgical outcomes. Numerical simulation (NS) helps understand hemodynamic phenomena, allowing to explore variations in physiological conditions, anatomy and tissue properties. Limitations due to simplifications and insufficient validation have hindered clinical use of this potentially powerful tool. The purpose of this study was to develop a numerical model of TCPC based on in vivo 4D-Flow-MRI measurements. An in vitro system was built with the potential objective of verifying numerical simulation.

## Methods

A 4D-Flow-MRI IRB-approved protocol was performed in a 2-year-old with hypoplastic left heart syndrome and extracardiac TCPC. Data was processed to generate flow trajectories and velocities. TCPC anatomy was isolated and converted to a virtual three-dimensional geometry. The latter was used to build a physical model (Nylon) using additive manufacturing and for NS of the in vivo case. Blood properties were defined (density 1050 kg/m3, viscosity 0.0035 Pa*s). Zero velocity was implemented at vessel walls, assumed to be rigid. An inflow (from in vivo measurements) of 0.63 and 0.87 L/min was applied at the inferior vena cava and superior vena cava, respectively, while zero-pressure was defined at the outlets (pulmonary arteries, PAs). Flow rate, velocity fields and streamlines were calculated. An in vitro system was set up, where blood mimicking fluid (BMF), 25 mL/s, circulated in through the physical model venae cavae using a continuous flow pump, and out through the PAs. This setup was imaged with the in vivo 4D-Flow-MRI protocol.

## Results

**Figure 1:**
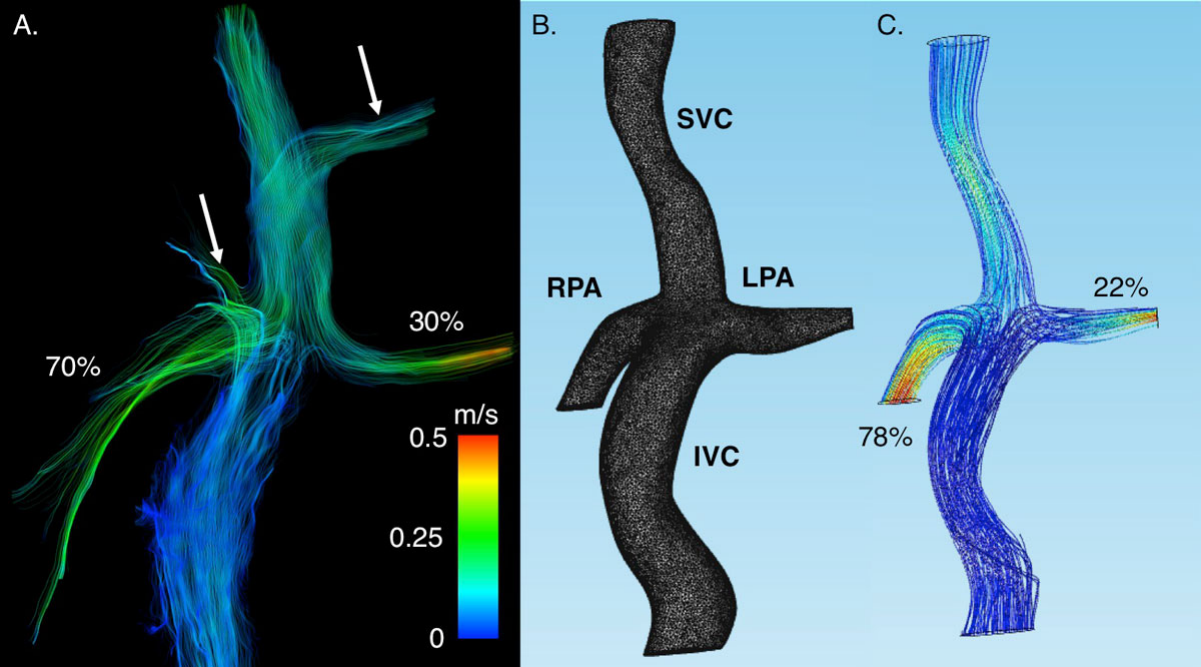
**4D-Flow-MRI was used to quantify flow hemodynamics in a 2 YO patient with a total cavo-pulmonary connection, and compared to a patient-specific numerical simulation**. **A**. In vivo flow distribution (streamlines) obtained from 4D-Flow-MRI (Ensight) (arrows show small vascular branches excluded in the numerical and physical models). **B**. Numerical mesh obtained from an angiogram generated from the 4D-Flow-MRI data (Mimics). **C**. Flow distribution (streamlines) obtained from the numerical simulation (Comsol). Similar results for maximum velocity were obtained in vivo (0.45 m/s) and in silico (0.56 m/s). In addition, good agreement was found between flow distribution (%) into the RPA and LPA in both the in vivo (70% vs 30%) and the numerical simulation (78% vs 22%) case.

**Figure 2:**
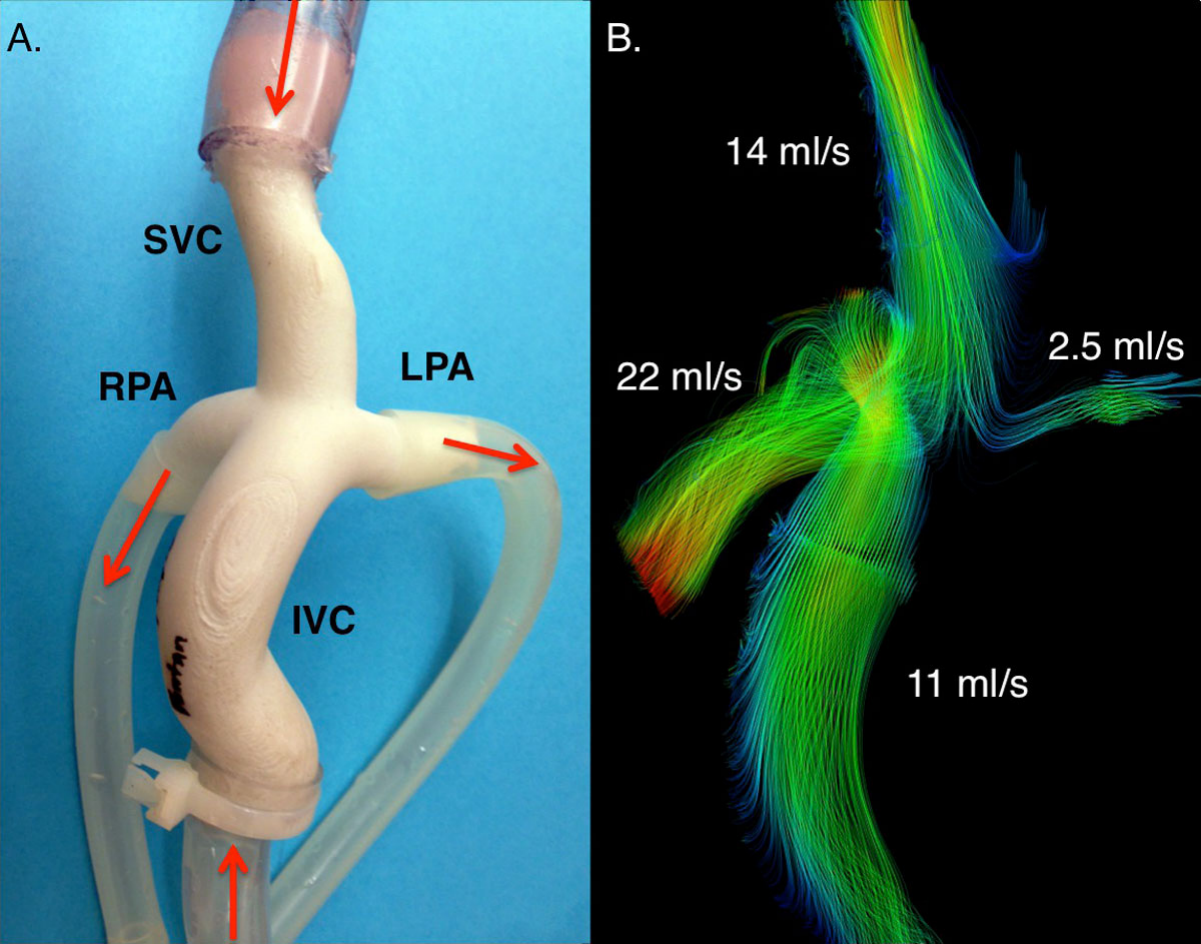
**Patient-specific in vitro model**. **A**. Patient-specific flow phantom constructed from 4D-Flow-MRI anatomical data using an additive manufacturing process (selective laser sintering). **B**. 4D-Flow-MRI was obtained for the in vitro model; flow distribution (streamlines) in the in vitro model show similar flow profiles as those seen in vivo and in the numerical simulations. Different flow distribution between RPA and LPA might be due to positioning of the tubing during MR scanning.

Results for in vivo 4D-Flow-MRI measurements and NS (Figure 1) show comparable velocities and pulmonary flow distribution. Results from in vitro 4D-Flow-MRI (Figure 2) demonstrate similar flow behavior.

## Conclusions

A numerical model was developed to simulate extracardiac TCPC, resulting in comparable velocities with respect to in vivo 4D-Flow-MRI measurements. Higher NS velocities were expected due to the rigidity of the vessel walls. Similar pulmonary flow distribution was found for both cases. An in vitro setup was developed to mimic and assess patient-specific TCPC hemodynamics while controlling various parameters, impossible in vivo. Results were qualitatively comparable. Future work focuses on improvement of in vitro design and on a quantitative assessment of flow and energy. The in vitro - NS combination offers both a numerical validation tool and a system where parameters are highly controlled, improving understanding and treatment of TCPC.

